# COVID-19 and Hyperinflammatory Syndrome in Children: Kawasaki Disease with Macrophage Activation Syndrome in Disguise?

**DOI:** 10.7759/cureus.9515

**Published:** 2020-08-01

**Authors:** Rohit S Loomba, Enrique G Villarreal, Saul Flores

**Affiliations:** 1 Cardiology, Advocate Children's Hospital, Chicago, USA; 2 Pediatrics, Tecnologico De Monterrey, Escuela De Medicina Y Ciencias De La Salud, Monterrey, MEX; 3 Cardiology, Texas Children's Hospital, Houston, USA

**Keywords:** pediatric inflammatory multisystem syndrome, covid-19, kawasaki disease, macrophage activation syndrome, pediatrics, cardiology, inflammation

## Abstract

A hyperinflammatory syndrome has been described in times of COVID-19 in children. In the setting of uncertainty due to a new virus, the so-called hyperinflammatory syndrome has been coined as a novel entity by some and is being referred to as pediatric inflammatory multisystem syndrome (PIMS). However, the characteristics of the syndrome resemble those of Kawasaki disease (KD), an inflammatory syndrome in children that can lead to coronary artery abnormalities due to a subsequent vasculitis. Furthermore, Kawasaki disease may occasionally trigger macrophage activation syndrome (MAS), a condition in which there is uncontrolled activation and proliferation of macrophages and other cell types, and could lead to multiorgan system dysfunction. This study provides a review of the data regarding COVID-19, Kawasaki disease, and macrophage activation syndrome to demonstrate the similarities and differences between the inflammatory syndrome seen with COVID-19 and KD. In addition, a framework for diagnosis and evaluation is provided that focuses on the pathway previously established for KD and MAS. The authors believe that based on current knowledge, KD treatment delays may carry deleterious effects in the near future for children with COVID-19-related Kawasaki disease.

## Introduction and background

As the COVID-19 pandemic continues globally, a hyperinflammatory syndrome in children has now been described [1,2]. This hyperinflammatory syndrome has been coined as a novel entity by some and is being referred to as pediatric inflammatory multisystem syndrome (PIMS). However, this entity nearly entirely overlaps with Kawasaki disease (KD), an inflammatory syndrome in children that can lead to coronary artery abnormalities due to a subsequent vasculitis.

KD, in both its typical and atypical presentations, has been described to be associated with viral illness, and in up to 2% of children, KD can be associated with macrophage activation syndrome (MAS) [3]. MAS is a condition in which there are uncontrolled activation and proliferation macrophages and other cell lines [4]. MAS in children has also been associated with juvenile idiopathic arthritis (JIA) [5]. MAS can lead to multiorgan system dysfunction and is associated with high morbidity and mortality.

The purpose of this study is to provide a review of KD and MAS in children with COVID-19 and to describe the differences and similarities between PIMS and KD with subsequent MAS in children with COVID-19. The understanding of these characteristics will be important in clinical management as there are already established, evidence-based management approaches to KD, which have been demonstrated to improve clinical outcomes, specifically in regard to coronary artery abnormalities.

## Review

COVID-19 and inflammatory response

A relatively small number of children have developed PIMS temporally related to the COVID-19 pandemic. PIMS has been characterized as the presentation of a child with high, persistent fever with elevated inflammatory markers such as neutrophilia, elevated C-reactive protein (CRP), and evidence of single or multiorgan dysfunction [6]. As stated, children with COVID-19 who require critical care admissions tend to have higher white blood cell counts, higher neutrophil counts, higher prothrombin time, higher D-dimer, lower albumin, higher transaminase levels, higher total bilirubin levels, higher lactate dehydrogenase, higher hypersensitive troponin I, and higher procalcitonin levels [7]. Not all pediatric patients with COVID-19 have elevated inflammatory markers but those who do, intuitively, appear to have more severe illness [8-10]. Similar findings have been described in adults where the laboratory markers that correlate with inflammation and/or organ dysfunction are associated with more severe illness and mortality [11].

A potential pathway by which severe acute respiratory syndrome coronavirus 2 (SARS-CoV-2) infects cells requires the angiotensin-converting enzyme 2 (ACE2) receptors and triggers pyroptosis and apoptosis resulting in the release of adenosine triphosphate and nucleic acids [12]. This then leads to CD4+ T lymphocytes activating into pathogenic T-helper cells [13]. This further incites a CD14+CD16+ monocyte response with high levels of circulating interleukin (IL)-6 [14]. While T lymphocytes and monocytes are upregulated, many of these cells may be ineffective in their ability to mount a successful host response to the virus. As previously described with SARS-CoV-1, the inflammatory state triggered by SARS-CoV-2 is triggered by a release of high levels of circulating cytokines, which can then damage end organs, particularly the lungs [15,16].

Viral illness and Kawasaki disease

The triggers of KD have not been entirely delineated. Nonetheless, viral illnesses seem to be a large contributing factor. Frequently, KD will develop two to three weeks after an acute viral illness. Viral illnesses that have been associated with KD include, but are not limited to, influenza enterovirus, adenovirus, parvovirus, rhinovirus, respiratory syncytial virus, varicella, Ebstein-Barr, measles, dengue, and coronavirus [17-30]. As mentioned, previous strains of coronavirus have been associated with KD.

Turnier et al. retrospectively reviewed their experience with 222 KD patients. Of these patients, 192 (86%) had a respiratory viral polymerase chain reaction performed. Of the 192 patients who were tested, 93 (41%) were found to have a concurrent viral illness, most frequently rhinovirus or enterovirus [31]. A study using the Taiwanese national database demonstrated that when compared to age and sex-matched children, those that were viral swab positive were 3.4 times more likely to have KD. Interestingly, when the viruses were individually analyzed, those with coronavirus were 7.8 times more likely to develop KD [3]. Furthermore, data from Japan and the United States, the Centers for Disease Control and Prevention and the national KD surveillance have reported that patients meeting KD for the first time had more rashes and less cervical lymphadenopathy compared to recurrent cases of KD. The median age for first-time KD patients was 24 months, whereas recurrent cases of KD were 42 months [32].

Seasonal variation has also been noted with KD. The time of year in which KD is more prevalent varies among countries and is likely related to different temporal prevalence in viruses [33-37].

Kawasaki disease and macrophage activation syndrome

A systematic review reported KD with MAS in children with a median age of 5.6 years and nearly 70% being male. Two-thirds of the patients were of Asian ethnicity. Of these patients, 77% had typical KD. These children often presented with neutropenia (median 1.8 x 10^9/L), thrombocytopenia (85.0 x 10^9/L), elevated erythrocyte sedimentation rate (64 mm/h), elevated C-reactive protein (7.3 mg/dl), elevated AST (32 U/L), elevated ALT (21 U/L), elevated lactate dehydrogenase (1,196 U/L), elevated ferritin (3,490 ng/mL), and elevated D-dimer (2,925 ng/mL). Of the 69 patients, 46% had coronary abnormalities and 13% of the 69 suffered mortality. All of the children received intravenous immunoglobulin (IVIG), nearly 90% of these children received steroids, nearly 50% received cyclosporine, and 39% received etoposide. In this review, of the 69 patients included, 74% met criteria for MAS secondary to juvenile idiopathic arthritis, the only clinical trigger for MAS for which there are such criteria [38].

The criteria to diagnose MAS in the setting of juvenile idiopathic arthritis are as follows: (1) fever; (2) ferritin greater than 684 ng/mL; (3) platelet count less than 181 x 10^9/L; (4) aspartate transaminase (AST) greater than 48 U/L; (5) triglycerides greater than 156 mg/dL; and (6) fibrinogen less than 360 mg/dL. All criteria must be met to receive the diagnosis. These criteria were created for MAS in the presence of juvenile idiopathic arthritis, hence, are not specific for KD, but have been applied for cases where there is suspicion. For instance, Verdoni and colleagues found that over half the patients with the necessary laboratory tests met criteria for JIA-related MAS in their cohort [2].

MAS generally requires anti-inflammatory therapy such as steroids. Mortality from JIA-related MAS has been reported in some cohorts to be approximately 40% [39]. The systematic review of KD-related MAS demonstrated mortality to be significantly lower [38]. Riphagen and colleagues reported 12% mortality, and Verdoni and colleagues reported 0% mortality in their cohort of ten patients [1,2]. The difference for the mortality rate difference remains unclear, however, patients with JIA tend to be chronically ill for longer periods of time and may carry other comorbidities as compared to children with KD.

Discussion

This review describes the inflammatory response to COVID-19, viral illness and its association with KD, and KD-associated MAS. As these concepts are synthesized it becomes intuitive that COVID-19 could trigger KD, which can then subsequently lead to MAS. MAS is characterized by a robust inflammatory response that may be associated with end-organ dysfunction. This is consistent with reports of a hyperinflammatory syndrome related to COVID-19, which is being referred to as PIMS. It will be important to identify the similarities and differences between PIMS and COVID-19 triggered KD and subsequent MAS. MAS is typically characterized by high fevers, liver and kidney injury, and bone marrow suppression with evidence of intravascular coagulation [40]. MAS is also associated with an elevation of inflammatory markers particularly an elevation of ferritin [41]. The high serum ferritin levels encountered in MAS reflect the presence of histiocytic hyperactivity. This is an important histopathologic finding that is shared by other entities. During this pandemic, another possible differential diagnosis that should be considered is hemophagocytic lymphohistiocytosis (HLH), a potentially fatal hyperinflammatory syndrome caused by excessive activation and expansion of T lymphocytes (predominantly CD8-positive) and macrophages that exhibit hemophagocytic activity [42]. In the acute phase of the process, HLH is almost indistinguishable from MAS. In fact, the clinical similarities between MAS and HLH are striking as both can be triggered in viral outbreaks. Though the final pathways are common between MAS and HLH, there are differences during the clinical course. Identifying these subtle differences is important as HLH carries a higher mortality rate compared to MAS. For patients with HLH, prompt initiation of treatment protocol that includes etoposide is essential, whereas the therapeutic approach in MAS depends on the clinical severity and would range from an increase in the dose of steroids to aggressive immunosuppressive medications [43-45].

Routine KD evaluation and management should be conducted. This includes serial echocardiogram, the administration of IVIG, and the administration of antiplatelet therapy. The mainstay of treatment for MAS is anti-inflammatory and immunosuppressive agents. This review highlights the importance of prompt identification of MAS, the need for close monitoring of these patients, and fast initiation of appropriate therapies.

As previously mentioned there are many similarities between KD and PIMS, but it is important to highlight some of the identified differences between these two entities identified thus far during the pandemic: (1) There appears to be more myocardial involvement in children with PIMS compared to KD; (2) there is lymphopenia in children with PIMS rather than normal or elevated lymphocytes as seen in children with KD; (3) PIMS appears to be more prevalent in older children with an average age of 7 years compared to children with KD where the average age is 3 years [2,46]; (4) additionally other differences between PIMS compared to KD-MAS are increased incidence of gastrointestinal system involvement and presence of rash [6]. Despite these differences, the outcomes of most children diagnosed with PIMS have been satisfactory with therapies similar to the ones utilized for KD.

Lastly, it is important for the field of pediatrics to determine whether PIMS is really a novel entity or SARS-CoV-2 is yet another trigger for KD and MAS as this perceived confusion may delay the application of proven therapies and impact patient outcomes. As recommended by several medical professional societies, information from patients with PIMS should be collected for further characterization of this entity, for a prompt diagnosis, and to determine optimal specific therapies.

## Conclusions

The recently described hyperinflammatory syndrome in the setting of COVID-19 is consistent with Kawasaki disease with subsequent macrophage activation syndrome. Clinical evaluation and management should thus follow the pathways developed for Kawasaki disease and macrophage activation syndrome. Delaying treatment for these known diagnoses may impact clinical outcome in children with COVID-19-related KD.
